# Development and validation of a predictive model for COPD: a multicenter study

**DOI:** 10.3389/fmed.2025.1615642

**Published:** 2025-09-09

**Authors:** Yaqin Wang, Yanyan Lv, Qiushuang Li, Ronghua Zhang, Bohua Yan, Hanrong Xue, Xiangqin Qian, Yu Yang, Kaiwen Ni, Jiayan Zhong, Xiang Meng, Rundi Gao, Zhen Wang

**Affiliations:** ^1^Department of Respiratory and Critical Care Medicine, The First Affiliated Hospital of Zhejiang Chinese Medical University (Zhejiang Provincial Hospital of Chinese Medicine), Hangzhou, China; ^2^The First School of Clinical Medicine, Zhejiang Chinese Medical University, Hangzhou, China; ^3^Center of Clinical Evaluation and Analysis, The First Affiliated Hospital of Zhejiang Chinese Medical University (Zhejiang Provincial Hospital of Chinese Medicine), Hangzhou, China; ^4^College of Pharmacy, Jinan University, Guangzhou, China; ^5^Guangdong Provincial Key Laboratory of Traditional Chinese Medicine Informatization, Jinan University, Guangzhou, China; ^6^Cancer Research Institution, Jinan University, Guangzhou, China; ^7^Department of Good Clinical Practice (GCP), Hospital of Chengdu University of Traditional Chinese Medicine, Chengdu, China; ^8^Department of Good Clinical Practice (GCP), Hospital of Jiangxi University of Traditional Chinese Medicine, Nanchang, China; ^9^Chronic Disease Management Department, Tao Zhuang Branch, The First People's Hospital of Jiashan County, Jiaxing, China; ^10^College of Information Science and Technology, Jinan University, Guangzhou, China; ^11^Department of Respiration, Wenzhou Hospital of Integrated Traditional Chinese and Western Medicine, Wenzhou, China

**Keywords:** chronic obstructive pulmonary disease, predictive model, risk factor, chronic obstructive pulmonary disease (COPD), clinical analysis

## Abstract

**Background:**

Chronic obstructive pulmonary disease (COPD) is the third leading cause of death globally and a major public health issue in China. This study aims to develop a COPD predictive model and conduct risk stratification for key indicators not included.

**Methods:**

We collected data from inpatients and outpatients with COPD and non-COPD who were hospitalized between January 2018 and December 2022 at three different hospitals. The data were divided into a training set and an internal validation set, using logistic regression to build a COPD predictive model and perform internal validation. External validation of the model was performed using data from two additional units for the period November 2019 to June 2022.

**Results:**

A total of 1,056 cases were included: 740 in the training set, 316 in the internal validation set, and 408 in the external validation set. Six risk factors were identified: age (OR = 1.05, 95% CI: 1.02–1.08), second-hand smoke exposure (OR = 8.27, 95% CI: 2.70–25.34), cough (OR = 23.52, 95% CI: 12.64–43.77), “occasional episodes of wheezing that are mild and do not interfere with sleep or activity” (OR = 6.06, 95% CI: 2.59–14.19), “bouts of wheezing that worsen with movement” (OR = 21.40, 95%CI: 10.32–44.37), and “persistent episodes of wheezing, occurring at rest, unable to lie down” (OR = 10.97, 95% CI: 1.02–118.28). The predictive model equation was: y = −5.920 + 0.047 (age) + 2.113 (smoke exposure) + 3.158 (cough) + 1.801 (wheezing 1) + 3.063 (wheezing 2) + 2.396 (wheezing 3). The model achieved 94.1% accuracy, 98.5% sensitivity, and 89.2% specificity, with an AUC of 0.976 (internal) and 0.691 (external). The critical cut-off value was 0.258.

**Conclusion:**

We have successfully developed a model for the diagnosis of COPD. The predictive model equation was: y = −5.920 + 0.047 (age) + 2.113 (smoke exposure) + 3.158 (cough) + 1.801 (wheezing 1) + 3.063 (wheezing 2) + 2.396 (wheezing 3).

## 1 Introduction

Chronic obstructive pulmonary disease (COPD) is a prevalent condition marked by persistent respiratory symptoms and airflow limitation. It is primarily characterized by chronic and often progressive airflow obstruction due to abnormalities in the airways (bronchitis) and/or alveoli (emphysema), resulting in chronic respiratory symptoms (dyspnea, cough, and sputum production) (1). According to the World Health Organization (WHO) (2), COPD ranks as the third leading cause of death globally, after ischemic heart disease and stroke. A high percentage of COPD cases remain undiagnosed. The GOLD 2023 guidelines discuss the impact of case-finding tools in improving COPD diagnosis rates, medical practices, and outcomes (1). In China, nearly 100 million people are affected by COPD, with the prevalence among those aged 40 years and older rising from 8.2% in 2007 to 13.7% in 2018 (3). COPD in China is characterized by high prevalence, morbidity, disability, mortality, and economic burden, along with low awareness (4). It has become one of the most prominent public health and medical problems in China in recent times.

The “gold standard” for diagnosing COPD relies on lung function testing. Despite standardized diagnosis and treatment protocols recommended by the Global Initiative for Chronic Obstructive Lung Disease (GOLD), most patients are not diagnosed until their symptoms become very pronounced. Consequently, by the time they seek medical attention, many patients already have impaired lung function. A nationwide epidemiological survey of COPD revealed that only 10% of respondents had undergone pulmonary function tests, and medication adherence among patients with COPD was as low as 11.7%. With the increasing prevalence of smoking in developing countries and increasing ageing in high-income countries, the incidence of COPD is projected to continue to rise over the next 40 years, with more than 5.4 million deaths from COPD and related diseases (5).

China is a country with a high prevalence of COPD, but due to its vast territory and numerous influencing factors, there is currently no widely used predictive tool to promote early diagnosis of COPD. This study aims to establish a more triage-oriented and applicable predictive model for COPD.

## 2 Methods

The research adhered to the Transparent Reporting of a Multivariable Prediction Model for Individual Prognosis or Diagnosis (TRIPOD) guidelines throughout the investigation and was conducted with the approval of the First Affiliated Hospital of Zhejiang Chinese Medical University (ethics number: 2019-KL-095-02). All patients who participated in the prospective study signed an informed consent form.

### 2.1 Study cohort and subgroups

Clinical data were retrospectively collected from inpatients and outpatients with COPD admitted to the First Affiliated Hospital of Zhejiang Chinese Medical University, the Affiliated Hospital of Jiangxi Chinese Medical University, and the Affiliated Hospital of Chengdu Chinese Medical University from January 2018 to December 2022. Data were also collected from non-COPD patients attending these hospitals during the same period. Additionally, the prospective inclusion of patients with COPD who visited the physical examination center of the First Affiliated Hospital of Zhejiang Chinese Medical University and the First People’s Hospital of Jiashan County (Tao Zhuang Branch) was performed from November 2019 to June 2022. The retrospective data were categorized into training and internal validation sets, while the prospective data were used as an external validation set.

The retrospective data used to establish the predictive model were obtained from inpatients and outpatients or patients undergoing health examinations who actively visited the respiratory medicine clinic. Since these were retrospective cases, the patients’ information had already been recorded in electronic medical records, including gender, age, and other details. For the non-COPD group, we invited two senior attending physicians to individually assess all non-COPD patients who visited the respiratory medicine clinic between January 2018 and December 2022. We excluded the following situations: (1) repeat visits; (2) acute exacerbation of the disease; (3) exclusion of patients who had undergone lung surgery or had lung tumors, interstitial lung disease, or other diseases that affect lung function or produce clinical symptoms similar to COPD. The non-COPD group excluded in this manner will serve as the control group. The same method was used in all three hospitals.

For the recruitment of the external validation population, investigators regularly arranged for two senior attending physicians to visit the health examination center and Taozhuang Health Center to conduct pulmonary function tests on individuals undergoing routine health screenings on a voluntary basis. If a patient’s pulmonary function met the diagnostic criteria for COPD according to the GOLD 2019 guidelines and the patient was willing to participate, we invited the patient to sign an informed consent form and complete a questionnaire.

### 2.2 Inclusion criteria

Retrospective cases: patients with a definitive diagnosis of COPD.

Prospective cases: According to the GOLD 2019 guidelines (6), the diagnosis of COPD is primarily based on a history of exposure to risk factors, symptoms, signs, and clinical data, such as pulmonary function tests. It involves excluding other diseases that can cause similar symptoms and persistent airflow limitation and conducting a comprehensive analysis. Lung function tests showing persistent airflow limitation are necessary to confirm the diagnosis of COPD, with an FEV1/FVC ratio of <70% after bronchodilator inhalation, clearly indicating persistent airflow limitation.

### 2.3 Data acquisition

The clinical information collected in this study included baseline characteristics, medical history, laboratory tests, and clinical symptoms:

Baseline characteristics (6): age, sex, Body Mass Index (BMI) (7–10), history of smoking, history of exposure to secondhand smoke, family history of respiratory disease, and a definite diagnosis of COPD.

Medical history (11–13): history of hypertension, hyperlipidemia, diabetes mellitus, stroke, and osteoporosis;

Laboratory tests: white blood cell count (WBC) (14), platelet count (PLT), hemoglobin level (Hb), neutrophil percentage (NE%), red blood cell count (RBC) (15), eosinophil count (Eos), apolipoprotein, uric acid (UA), fasting blood glucose (FBG), and pulmonary function;

Clinical symptoms: cough, cough sputum (divided into three categories based on the amount of cough sputum: “0” indicates “no sputum or little sputum (sputum volume <50 mL)”; “1” indicates “moderate amount of sputum (sputum volume of 50–100 mL)”; “2” indicates “a lot of sputum (sputum volume >100 mL)”), and wheezing (divided into four categories based on the degree of wheezing: “0” indicates ‘“no significant wheezing”’; ‘1’ indicates ‘persistent episodes of wheezing, occurring at rest, unable to lie down’; ‘2’ indicates ‘wheezing episodes that worsen with movement’; and ‘3’ indicates ‘persistent episodes of wheezing, occurring at rest, unable to lie down’).

### 2.4 Sample size estimation, culling, and missing value treatment

According to the events per variable (EPV) principle, the minimum sample size required to build a predictive model is 10 times the number of variables included (16). Samples with > 10% missing values were excluded, and multiple interpolations were used to fill in the missing values.

### 2.5 Statistical analysis

A predictive model was developed and validated based on the TRIPOD guidelines (17). SAS (version 9.4) was used to randomly divide the retrospective data into a training set and an internal validation set at a ratio of 7:3. The training set, internal validation set, and external validation set were used for modeling, internal validation of the model, and external validation of the model, respectively.

IBM SPSS Statistics 26 was used to statistically analyze the data.

A systematic review (18) shows no performance benefit of machine learning over logistic regression for clinical prediction models. Furthermore, machine learning carries the risk of overfitting; thus, this study uses logistic regression to establish a prediction model.

**Figure 1:**
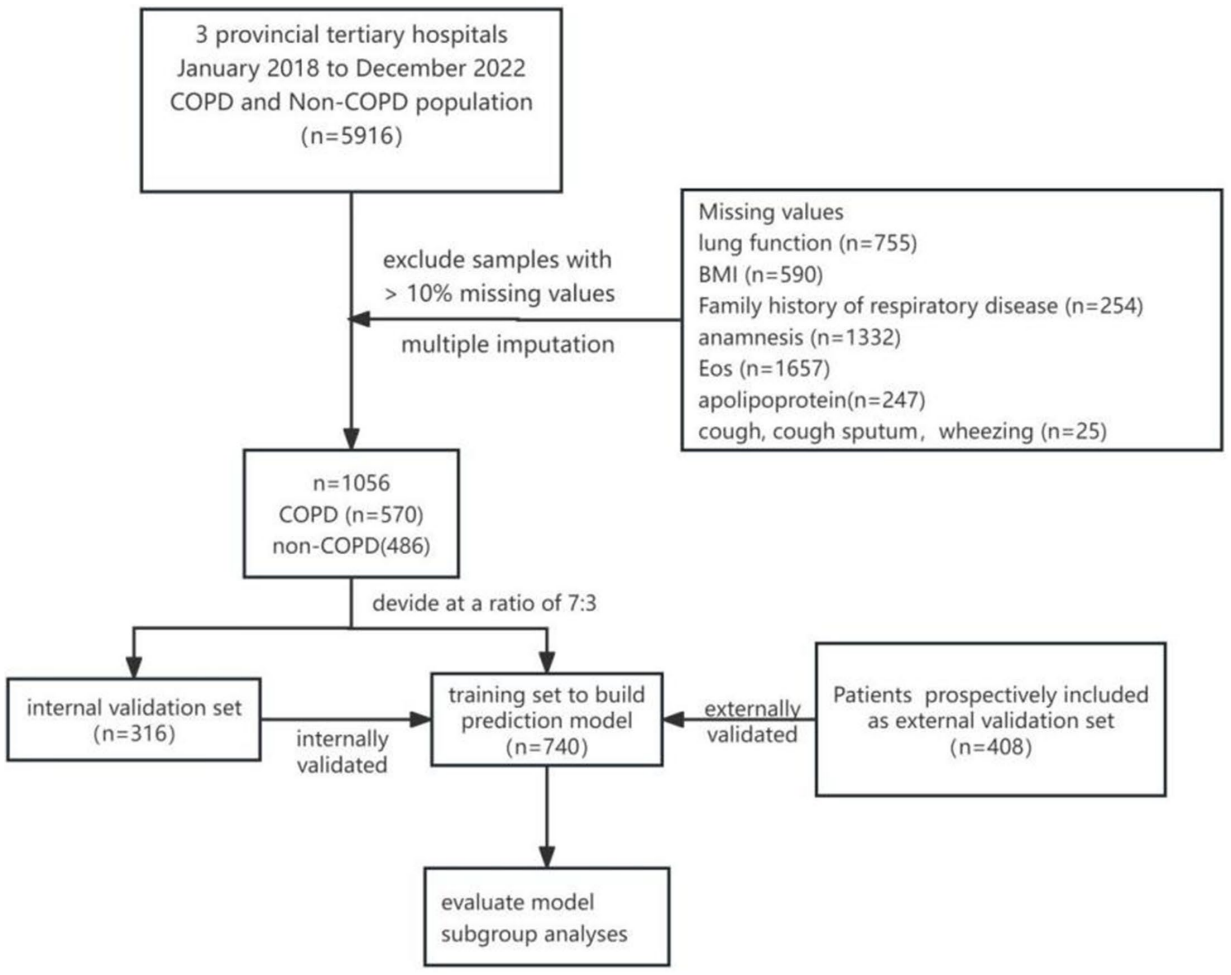
A technology roadmap of the this study.

First, in the training set, all independent variables were screened using univariate logistic regression to identify independent risk factors. All independent risk factors were then included in a multivariate logistic regression analysis, and the final predictive model was obtained through backward stepwise regression. This model was then applied to the internal and external validation sets for validation. The model’s performance was assessed by calculating the AUC under the ROC curve. Additional evaluation indices included accuracy, sensitivity, specificity, negative predictive value (NPV), and positive predictive value (PPV). A nomogram was constructed, and the calibration of the model was assessed using calibration curves. Finally, risk stratification of the model was performed in subgroups based on smoking history, BMI, pack-years of smoking, smoking cessation history, age at cessation, and EOS ratio (Eos%) (Figure 1).

## 3 Results

### 3.1 Study sample

In this study, 5,916 individuals were initially retrospectively included. After excluding 755 cases with pulmonary function deficiency, 590 cases with BMI deficiency, 254 cases with a family history of respiratory disease, 1,332 cases with a history of hypertension, hyperlipidemia, diabetes mellitus, and stroke, 1,657 cases with EOS deficiency, 247 cases with apolipoprotein deficiency, and 25 cases with coughing, sputum, and wheezing deficiencies, a final sample of 1,056 participants were included for analysis.

Multiple imputation was performed on the 1,056 samples and averaged across five imputations. There were no statistically significant differences in the data before and after interpolation (*p* > 0.05), as shown in Table 1.

**Table 1:** Sensitivity analysis before and after missing value interpolation.

Characteristics	Missing values *N* (%)	Interpolation	Interpolation	Statistic	*p*-value
PLT count M(Q1, Q3)	1 (0.09)	205.00 (162.00, 254.00)	204.50 (162.00, 254.00)	*Z* = 0.018	0.986
Hb content Mean±SD	1 (0.09)	128.44 ± 18.89	128.41 ± 18.91	*t* = 0.04	0.970
NE % Mean±SD	3 (0.28)	61.94 ± 18.70	61.91 ± 18.76	*t* = 0.04	0.969
FBG Mean±SD	66 (6.25)	5.44 ± 1.54	5.45 ± 1.58	*t* = −0.18	0.859
Second-hand smoke *n* (%)	48 (4.55)			*χ^2^* = 0.006	0.938

Additionally, a total of 408 patients with COPD with complete data were prospectively included in this study.

### 3.2 Establishment and validation of the prediction model for COPD

#### 3.2.1 Comparison of equivalence between the training set and internal validation set

Using SAS 9.4, all 1,056 cases were randomly split with a random seed into a training set comprising 70% (*n* = 740) and an internal validation set comprising 30% (*n* = 316). There were no significant differences between the two groups in terms of age, sex, BMI, history of hypertension, hyperlipidemia, diabetes mellitus, stroke, osteoporosis, WBC count, PLT count, Hb content, neutrophil ratio, RBC count, apolipoprotein A, FBG, EOS count, history of smoking, exposure to secondhand smoke, family history of respiratory diseases, cough, sputum, and wheezing (Table 2).

**Table 2:** Equitability on training set and internal validation set.

Characteristics *N* (%)	General collection (*n* = 1,056)	Groups		*T/χ2/Z*	*p*-value
		Internal validation set (*n* = 316)	Training set (*n* = 740)		
Age, Mean ± SD	62.86 ± 14.83	62.75 ± 15.11	62.91 ± 14.72	*t* = −0.16	0.871
Sex				*χ^2^* = 0.396	0.529
Male	670 (63.45)	205 (64.87)	465 (62.84)		
Female	386 (36.55)	111 (35.13)	275 (37.16)		
BMI, Mean ± SD	23.39 ± 4.67	23.12 ± 4.06	23.50 ± 4.91	*t* = −1.29	0.199
BMI				*χ^2^* = 0.304	0.859
<18.5	99 (9.38)	32 (10.13)	67 (9.05)		
18.5–24	544 (51.52)	161 (50.95)	383 (51.76)		
≥24	413 (39.11)	123 (38.92)	290 (39.19)		
History of hypertension				*χ^2^* = 0.079	0.778
Not have	715 (67.71)	212 (67.09)	503 (67.97)		
There are	341 (32.29)	104 (32.91)	237 (32.03)		
History of hyperlipidemia				*χ^2^* = 1.843	0.175
No	1,037 (98.20)	313 (99.05)	724 (97.84)		
Yes	19 (1.80)	3 (0.95)	16 (2.16)		
History of diabetes				*χ^2^* = 0.079	0.778
No	715 (67.71)	212 (67.09)	503 (67.97)		
Yes	341 (32.29)	104 (32.91)	237 (32.03)		
History of stroke				*χ^2^* = 0.354	0.552
No	1,017 (96.31)	306 (96.84)	711 (96.08)		
Yes	39 (3.69)	10 (3.16)	29 (3.92)		
History of osteoporosis				*χ^2^* = 1.841	0.175
No	1,038 (98.30)	308 (97.47)	730 (98.65)		
Yes	18 (1.70)	8 (2.53)	10 (1.35)		
Uric acid, Mean ± SD	304.03 ± 89.93	302.49 ± 88.11	304.69 ± 90.75	*t* = −0.36	0.715
WBC count, M (Q1, Q3)	5.90 (4.70, 7.70)	6.00 (4.90, 7.55)	5.90 (4.70, 7.70)	*Z* = 0.655	0.513
PLT count, M (Q1, Q3)	204.50 (162.00, 254.00)	207.00 (164.00, 253.50)	203.50 (161.50, 255.00)	*Z* = 0.507	0.612
Hb content, Mean ± SD	128.41 ± 18.91	129.84 ± 17.88	127.81 ± 19.32	*t* = 1.60	0.110
NE %, Mean ± SD	61.91 ± 18.76	61.23 ± 19.32	62.20 ± 18.53	t = −0.78	0.438
erythrocyte count, M (Q1, Q3)	4.20 (3.80, 4.60)	4.20 (3.90, 4.70)	4.10 (3.80, 4.60)	*Z* = 1.925	0.054
Apolipoprotein A, M (Q1, Q3)	1.30 (1.10, 1.50)	1.30 (1.10, 1.50)	1.30 (1.10, 1.50)	*Z* = 0.258	0.796
FBG, Mean ± SD	5.45 ± 1.58	5.55 ± 1.61	5.41 ± 1.56	*t* = 1.26	0.207
EOS count, M (Q1, Q3)	0.10 (0.00, 0.45)	0.11 (0.00, 0.40)	0.10 (0.00, 0.47)	*Z* = 0.323	0.747
Cigarette smoking				*χ^2^* = 0.112	0.738
No	766 (72.54)	227 (71.84)	539 (72.84)		
Yes	290 (27.46)	89 (28.16)	201 (27.16)		
Second-hand smoke exposure				*χ^2^* = 1.037	0.308
No	897 (84.94)	263 (83.23)	634 (85.68)		
Yes	159 (15.06)	53 (16.77)	106 (14.32)		
Family history of respiratory disease				-	0.509
No	1,054 (99.81)	315 (99.68)	739 (99.86)		
Yes	2 (0.19)	1 (0.32)	1 (0.14)		
Cough				*χ^2^* = 0.089	0.765
No	502 (47.54)	148 (46.84)	354 (47.84)		
Yes	554 (52.46)	168 (53.16)	386 (52.16)		
Phlegm				*Z* = 1.355	0.176
0	522 (49.43)	144 (45.57)	378 (51.08)		
1	208 (19.70)	70 (22.15)	138 (18.65)		
2	326 (30.87)	102 (32.28)	224 (30.27)		
Wheezing				*Z* = 1.085	0.278
0	539 (51.04)	148 (46.84)	391 (52.84)		
1	178 (16.86)	66 (20.89)	112 (15.14)		
2	321 (30.40)	99 (31.33)	222 (30.00)		
3	18 (1.70)	3 (0.95)	15 (2.03)		
COPD				*χ^2^* = 2.376	0.123
No	486 (46.02)	134 (42.41)	352 (47.57)		
Yes	570 (53.98)	182 (57.59)	388 (52.43)		

*t* refers to t-test, *χ*^2^ refers to chi-square test, *Z* refers to Mann–Whitney U test, SD refers to standard deviation, M refers to median, Q1 refers to 1st quartile, and Q3 refers to 3rd quartile.

#### 3.2.2 Basic characteristics of COPD and non-COPD patients in the training set

In the training set of 740 samples, there were 388 patients with COPD and 352 non-COPD patients. A comparison between the two groups revealed that patients with COPD exhibited higher levels of age, male sex, history of hypertension, history of diabetes mellitus, uric acid levels, WBC count, NE%, history of cigarette smoking, exposure to second-hand smoke, presence of cough, phlegm, and wheezing compared to non-COPD patients. However, patients with COPD had lower levels of BMI and apolipoprotein A compared to non-COPD patients (Table 3).

**Table 3:** Basic characteristics of COPD and non-COPD in the training set.

Characteristics *n* (%)	Total sample (*n* = 740)	Whether COPD		*t/χ^2^/Z*	*p*-value
		No (*n* = 352)	Yes (*n* = 388)		
Age, Mean ± SD	62.91 ± 14.72	54.69 ± 14.72	70.37 ± 10.02	*t* = −16.77	<0.001
Sex				*χ^2^* = 124.288	<0.001
Male	465 (62.84)	148 (42.05)	317 (81.70)		
Female	275 (37.16)	204 (57.95)	71 (18.30)		
BMI, Mean ± SD	23.50 ± 4.91	24.04 ± 4.94	23.01 ± 4.84	*t* = 2.87	0.004
BMI				*χ^2^* = 20.200	<0.001
<18.5	67 (9.05)	15 (4.26)	52 (13.40)		
18.5–24	383 (51.76)	184 (52.27)	199 (51.29)		
≥24	290 (39.19)	153 (43.47)	137 (35.31)		
History of hypertension				*χ^2^* = 17.790	<0.001
No	503 (67.97)	266 (75.57)	237 (61.08)		
Yes	237 (32.03)	86 (24.43)	151 (38.92)		
History of hyperlipidemia				*χ^2^* = 0.494	0.482
No	724 (97.84)	343 (97.44)	381 (98.20)		
Yes	16 (2.16)	9 (2.56)	7 (1.80)		
History of diabetes				*χ^2^* = 17.790	<0.001
No	503 (67.97)	266 (75.57)	237 (61.08)		
Yes	237 (32.03)	86 (24.43)	151 (38.92)		
History of stroke				*χ^2^* = 0.463	0.496
No	711 (96.08)	340 (96.59)	371 (95.62)		
Yes	29 (3.92)	12 (3.41)	17 (4.38)		
History of osteoporosis				–	0.111
No	730 (98.65)	350 (99.43)	380 (97.94)		
Yes	10 (1.35)	2 (0.57)	8 (2.06)		
Uric acid, Mean ± SD	304.69 ± 90.75	295.61 ± 80.93	312.94 ± 98.20	*t* = −2.63	0.009
WBC count, M (Q1, Q3)	5.90 (4.70, 7.70)	5.55 (4.35, 6.70)	6.30 (5.10, 8.30)	*Z* = −5.975	<0.001
PLT count, M (Q1, Q3)	203.50 (161.50, 255.00)	209.50 (162.00, 263.50)	196.00 (161.00,246.00)	*Z* = 1.412	0.158
Hb content, Mean ± SD	127.81 ± 19.32	127.40 ± 19.15	128.18 ± 19.48	*t* = −0.55	0.585
NE%, Mean ± SD	62.20 ± 18.53	60.37 ± 16.11	63.86 ± 20.35	*t* = −2.60	0.010
RBC count, M (Q1, Q3)	4.10 (3.80, 4.60)	4.10 (3.80, 4.50)	4.10 (3.80, 4.70)	*Z* = −0.240	0.810
Apolipoprotein A, M (Q1, Q3)	1.30 (1.10, 1.50)	1.40 (1.30, 1.60)	1.20 (1.10, 1.40)	*Z* = 8.590	<0.001
FBG, Mean ± SD	5.41 ± 1.56	5.34 ± 1.27	5.48 ± 1.78	*t* = −1.21	0.228
EOS count, M (Q1, Q3)	0.10 (0.00, 0.47)	0.10 (0.00, 0.40)	0.10 (0.00, 0.50)	*Z* = −1.719	0.086
Cigarette smoking				*χ*^2^ = 128.920	<0.001
No	539 (72.84)	325 (92.33)	214 (55.15)		
Yes	201 (27.16)	27 (7.67)	174 (44.85)		
Second-hand smoke				*χ*^2^ = 95.141	<0.001
No	634 (85.68)	348 (98.86)	286 (73.71)		
Yes	106 (14.32)	4 (1.14)	102 (26.29)		
Family history of respiratory disease				–	1.000
No	739 (99.86)	352 (100.00)	387 (99.74)		
Yes	1 (0.14)	0 (0.00)	1 (0.26)		
Cough				*χ*^2^ = 479.547	<0.001
No	354 (47.84)	317 (90.06)	37 (9.54)		
Yes	386 (52.16)	35 (9.94)	351 (90.46)		
Phlegm				*Z* = −19.407	<0.001
0	378 (51.08)	317 (90.06)	61 (15.72)		
1	138 (18.65)	17 (4.83)	121 (31.19)		
2	224 (30.27)	18 (5.11)	206 (53.09)		
Wheezing				*Z* = −19.690	<0.001
0	391 (52.84)	325 (92.33)	66 (17.01)		
1	112 (15.14)	11 (3.13)	101 (26.03)		
2	222 (30.00)	15 (4.26)	207 (53.35)		
3	15 (2.03)	1 (0.28)	14 (3.61)		

*t* refers to t-test, *χ*^2^ refers to chi-square test, *Z* refers to Mann–Whitney U test, −: Fisher’s exact test, SD refers to standard deviation, M refers to median, Q1 refers to 1st quartile, Q3 refers to 3rd quartile.

#### 3.2.3 Univariate logistic regression in the training set

The univariate logistic regression analysis of the training set revealed that the following factors were independent risk factors for COPD: age, sex, BMI, history of hypertension, history of diabetes, uric acid levels, WBC count, NE%, RBC count, apolipoprotein A, history of cigarette smoking, exposure to secondhand smoke, presence of cough, sputum, and wheezing (Table 4).

**Table 4:** Training set one-factor logistics regression.

Characteristics	*β*	S. E	Wald	OR (95% CI)	*p*-value
Age	0.105	0.009	151.315	1.11 (1.09–1.13)	<0.001
Sex					
Male				Reference point	
Female	−1.817	0.170	114.263	0.16 (0.12–0.23)	<0.001
BMI					
<18.5	1.165	0.310	14.081	3.21 (1.74–5.89)	<0.001
18.5–24				Reference point	
≥24	−0.189	0.156	1.468	0.83 (0.61–1.12)	0.226
History of hypertension					
No				Reference point	
Yes	0.678	0.162	17.544	1.97 (1.43–2.71)	<0.001
History of hyperlipidemia					
No				Reference point	
Yes	−0.356	0.509	0.488	0.70 (0.26–1.90)	0.485
History of diabetes					
No				Reference point	
Yes	0.678	0.162	17.544	1.97 (1.43–2.71)	<0.001
History of stroke					
No				Reference point	
Yes	0.261	0.384	0.461	1.30 (0.61–2.76)	0.497
History of osteoporosis					
No				Reference point	
Yes	1.304	0.794	2.697	3.68 (0.78–17.47)	0.101
Uric acid	0.002	0.001	6.657	1.01 (1.01–1.01)	0.010
WBC count	0.183	0.033	31.308	1.20 (1.13–1.28)	<0.001
PLT count	−0.001	0.001	0.311	1.00 (1.00–1.00)	0.577
Hb content	0.002	0.004	0.299	1.00 (0.99–1.01)	0.584
Ne%	0.010	0.004	6.441	1.01 (1.01–1.02)	0.011
RBC count	0.077	0.033	5.601	1.08 (1.01–1.15)	0.018
Apolipoprotein A	−1.659	0.283	34.295	0.19 (0.11–0.33)	<0.001
FBG	0.057	0.048	1.397	1.06 (0.96–1.16)	0.237
EOS count	0.060	0.043	1.920	1.06 (0.98–1.15)	0.166
Cigarette smoking					
No				Reference point	
Yes	2.281	0.225	102.966	9.79 (6.30–15.21)	<0.001
Second-hand smoke					
No				Reference point	
Yes	3.435	0.516	44.326	31.03 (11.29–85.29)	<0.001
Family history of respiratory disease					
No				Reference point	
Yes	12.112	447.33	0.001	–	0.978
Cough					
No				Reference point	
Yes	4.453	0.248	321.953	85.92 (52.82–139.75)	<0.001
Phlegm					
0				Reference point	
1	3.611	0.294	150.474	36.99 (20.77–65.86)	<0.001
2	4.085	0.283	208.756	59.47 (34.17–103.51)	<0.001
Wheezing symptoms					
0				Reference point	
1	3.811	0.345	122.034	45.21 (22.99–88.91)	<0.001
2	4.219	0.300	198.376	67.94 (37.77–122.20)	<0.001
3	4.233	1.044	16.447	68.92 (8.91–533.09)	<0.001

### 3.3 COPD predictive model and nomogram

All independent risk factors identified in the univariate logistic regression were included in a multivariate logistic regression analysis using backward stepwise regression to establish a predictive model for COPD. The final factors included in the predictive model were age, secondhand smoke exposure, coughing, and wheezing (Table 5).

**Table 5:** Variables included in final model.

Characteristics	*β*	S. E	Wald	OR (95% CI)	*p*-value
Constant	−5.920	0.863	47.040		<0.001
Age	0.047	0.013	13.177	1.05 (1.02–1.08)	<0.001
Second-hand smoke					
No				Reference point	
Yes	2.113	0.571	13.694	8.27 (2.70–25.34)	<0.001
Cough					
No				Reference point	
Yes	3.158	0.317	99.290	23.52 (12.64–43.77)	<0.001
Wheezing					
0				Reference point	
1	1.801	0.434	17.216	6.06 (2.59–14.19)	<0.001
2	3.063	0.372	67.755	21.40 (10.32–44.37)	<0.001
3	2.396	1.213	3.900	10.97 (1.02–118.28)	0.048

The risk of COPD increased by 0.05-fold for each additional year of age (OR = 1.05, 95%CI: 1.02–1.08). Individuals exposed to secondhand smoke had a 7.27-fold higher risk of COPD compared to those without such exposure (OR = 8.27, 95%CI: 2.70–25.34). Patients with coughing showed a 22.52-fold increase in the risk of COPD (OR = 23.52, 95%CI: 12.64–43.77) compared to those without this symptom. Different levels of wheezing symptoms also indicated varying levels of COPD risk.

The formula for the final model is as follows: y = −5.920 + 0.047 (age) + 2.113 (history of secondhand smoke) + 3.158 (having cough) + 1.801 (wheezing symptom 1) + 3.063 (wheezing symptom 2) + 2.396 (wheezing symptom 3), logit(p) = $\frac{e^{y}}{1+e^{y}}$ , where p represents probability and logit(p) is distributed between 0 and 1. A higher logit(p) indicates a greater risk of COPD.

**Figure 2:**
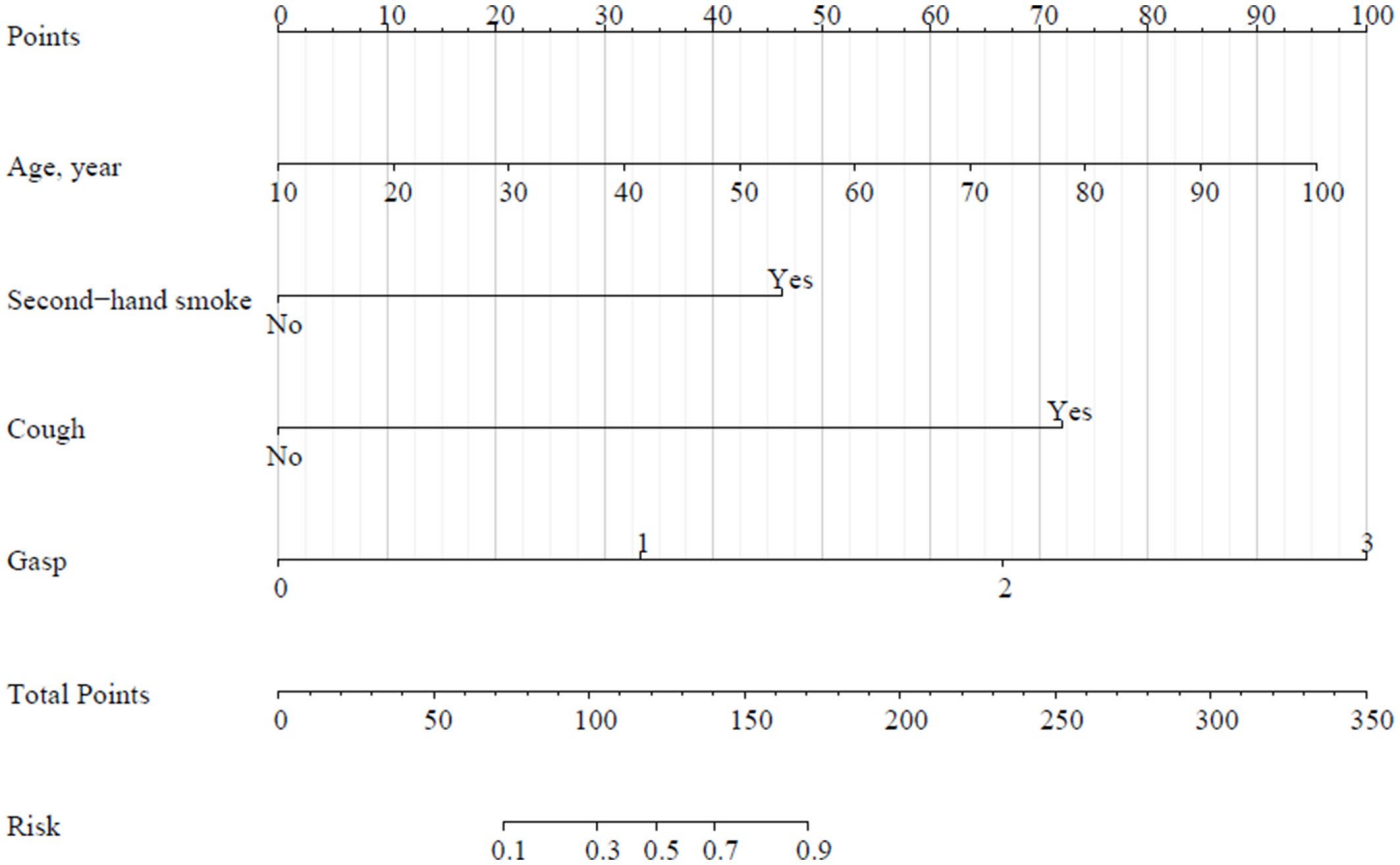
Nomogram of COPD prediction model.

Using R 4.1.3, a nomogram was plotted where each diagnostic factor corresponds to a score (also called a point). The scores from these factors were summed to obtain a total score (total points), which correlates with the corresponding risk of COPD (Figure 2).

### 3.4 Characterization of the external validation set

The external validation set comprised a total of 408 samples, consisting of 141 patients with COPD and 267 non-COPD individuals (Table 6).

**Table 6:** Baseline comparison of external validation set.

Characteristics *N* (%)	General collection (*n* = 408)	Group		*T/χ2/z*	*p*-value
		Non-copd (*n* = 267)	Copd (*n* = 141)		
Age, Mean ± SD	66.71 ± 18.50	64.08 ± 21.65	71.70 ± 8.11	t = −5.12	<0.001
Sex				*χ*^2^ = 11.684	<0.001
Male	280 (68.63)	168 (62.92)	112 (79.43)		
Female	128 (31.37)	99 (37.08)	29 (20.57)		
BMI, Mean ± SD	24.29 ± 12.37	24.75 ± 14.91	23.42 ± 4.73	*t* = 1.33	0.184
Second-hand smoke				*χ*^2^ = 3.662	0.056
No	202 (49.51)	123 (46.07)	79 (56.03)		
Yes	206 (50.49)	144 (53.93)	62 (43.97)		
Cough				*χ*^2^ = 7.146	0.008
No	127 (31.13)	95 (35.58)	32 (22.70)		
Yes	281 (68.87)	172 (64.42)	109 (77.30)		
Wheezing symptoms				–	<0.001
0	238 (58.33)	190 (71.16)	48 (34.04)		
1	95 (23.28)	57 (21.35)	38 (26.95)		
2	68 (16.67)	16 (5.99)	52 (36.88)		
3	7 (1.72)	4 (1.50)	3 (2.13)		
WBC count, M (Q1, Q3)	5.35 (4.50, 6.40)	5.70 (4.70, 6.50)	5.00 (4.50, 6.00)	*Z* = −0.877	0.381
PLT count, M (Q1, Q3)	182.00 (153.00, 227.00)	161.50 (147.00, 206.00)	224.0 (174.00, 252.00)	*Z* = 2.852	0.004
RBC count, Mean ± SD	4.52 ± 0.58	4.51 ± 0.55	4.53 ± 0.62	*t* = −0.15	0.879
EOS count, M (Q1, Q3)	0.32 (0.10, 1.70)	1.00 (0.10, 1.90)	0.14 (0.10, 0.20)	*Z* = −2.062	0.039
Cigarette smoking				*χ*^2^ = 1.545	0.214
No	192 (55.17)	122 (52.81)	70 (59.83)		
Yes	156 (44.83)	109 (47.19)	47 (40.17)		
Phlegm				*χ*^2^ = 31.179	<0.001
0	142 (35.24)	116 (44.11)	26 (18.57)		
1	183 (45.41)	111 (42.21)	72 (51.43)		
2	78 (19.35)	36 (13.69)	42 (30.00)		

### 3.5 Validation of prediction model

The model was tested on both internal and external validation sets to assess its discrimination and calibration.

#### 3.5.1 Discrimination test

**Figure 3:**
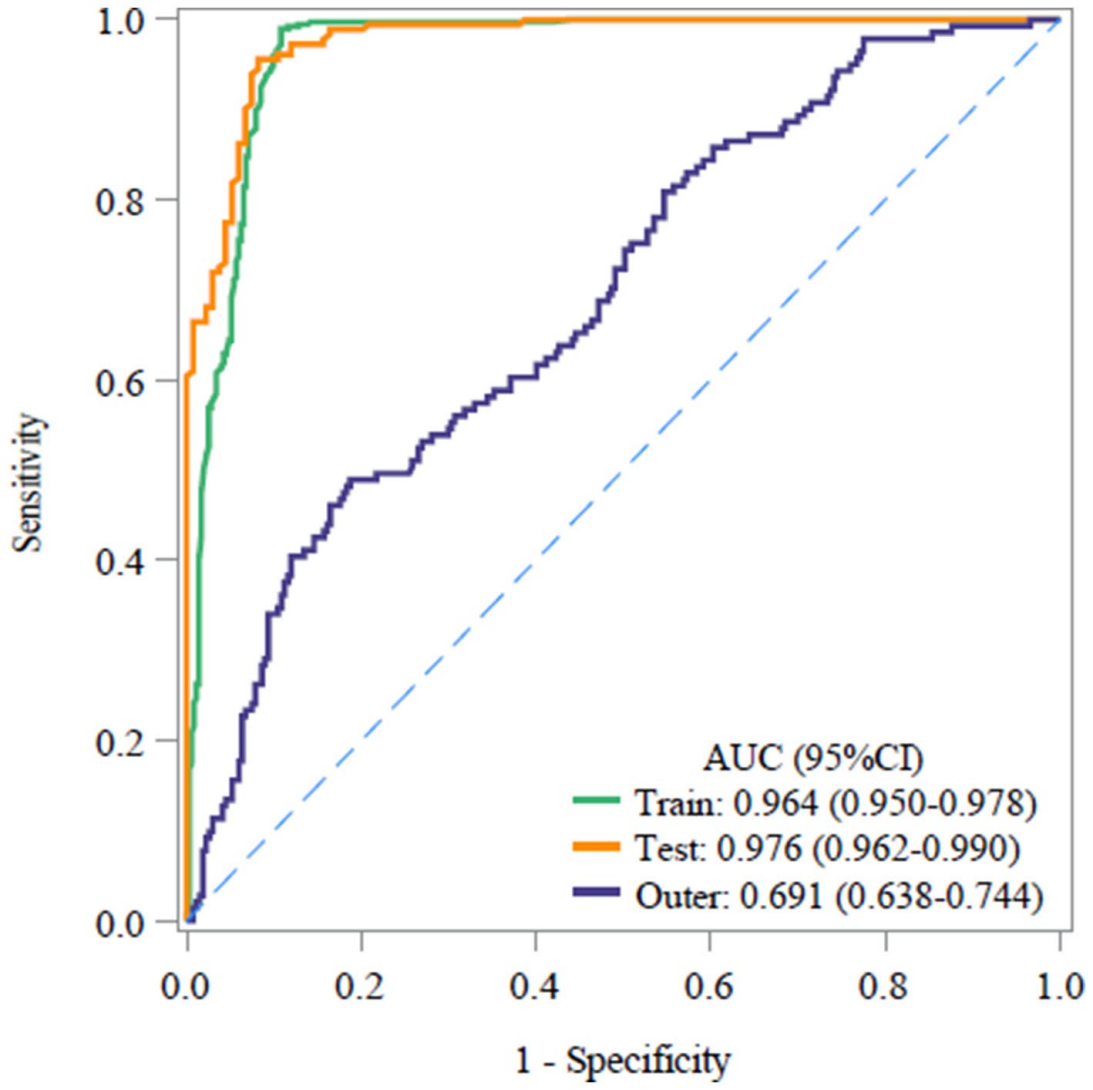
ROC curves of the model and its internal and external validation sets.

In the COPD prediction model, the area under the curve (AUC) for the training set was 0.964 (95% CI: 0.950–0.978), with an accuracy of 94.1%, a sensitivity of 98.5% and a specificity of 89.2%. For the internal validation set, the AUC was 0.976 (95% CI: 0.962–0.990), with an accuracy of 93.4%, a sensitivity of 96.2%, and a specificity of 89.6%. These results indicate that the model effectively discriminates samples from the same source and demonstrates excellent predictive capability for assessing the risk of COPD (Figure 3).

The AUC of the external validation set was 0.691 (95% CI: 0.962–0.990), with an accuracy of 49.3%, a sensitivity of 94.3%, and a specificity of 25.5%. Additionally, it demonstrated a PPV of 92.6% and an NPV of 94.5%, indicating a lack of generalization power for the model (Table 7).

**Table 7:** Results of predictive model and internal and external validation.

Characteristics	Training set	Internal validation set	External validation set
Cutoff	0.258	0.258	0.258
AUC (95% CI)	0.964 (0.950–0.978)	0.976 (0.962–0.990)	0.691 (0.638–0.744)
Accuracy (95% CI)	0.941 (0.921–0.956)	0.934 (0.900–0.958)	0.493 (0.443–0.542)
Sensitivity (95% CI)	0.985 (0.967–0.994)	0.962 (0.922–0.984)	0.943 (0.891–0.975)
Specificity (95% CI)	0.892 (0.855–0.922)	0.896 (0.831–0.942)	0.255 (0.204–0.311)
NPV (95% CI)	0.981 (0.960–0.993)	0.945 (0.890–0.978)	0.895 (0.803–0.953)
PPV (95% CI)	0.910 (0.878–0.935)	0.926 (0.879–0.959)	0.401 (0.347–0.456)

The cutoff value of the predictive model was 0.258, meaning that when logit(p) was > 0.258, the individual can be diagnosed with COPD according to the model; otherwise, they are not diagnosed with COPD.

#### 3.5.2 Calibration test

**Figure 4:**
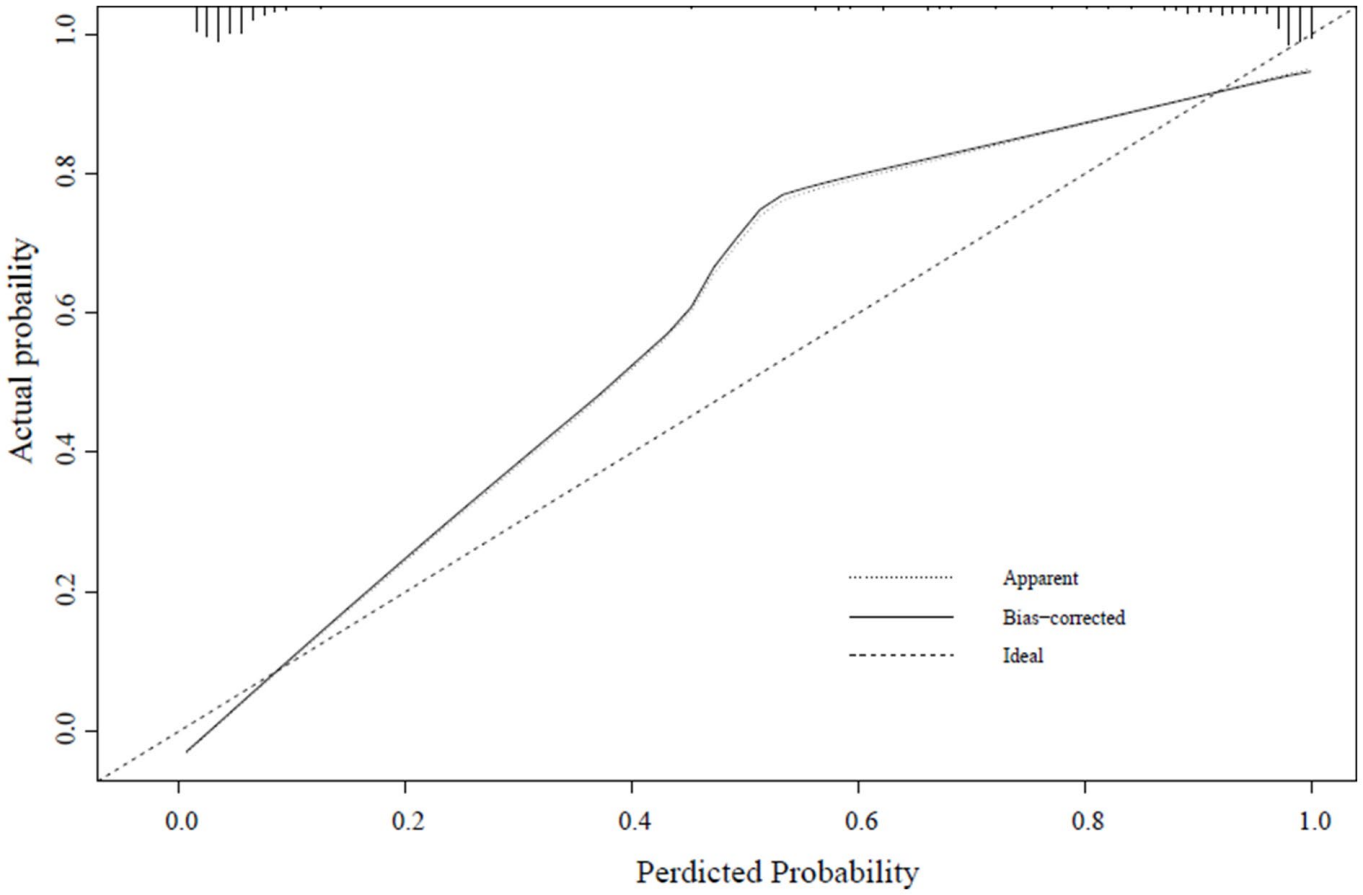
Training set calibration curve.

**Figure 5:**
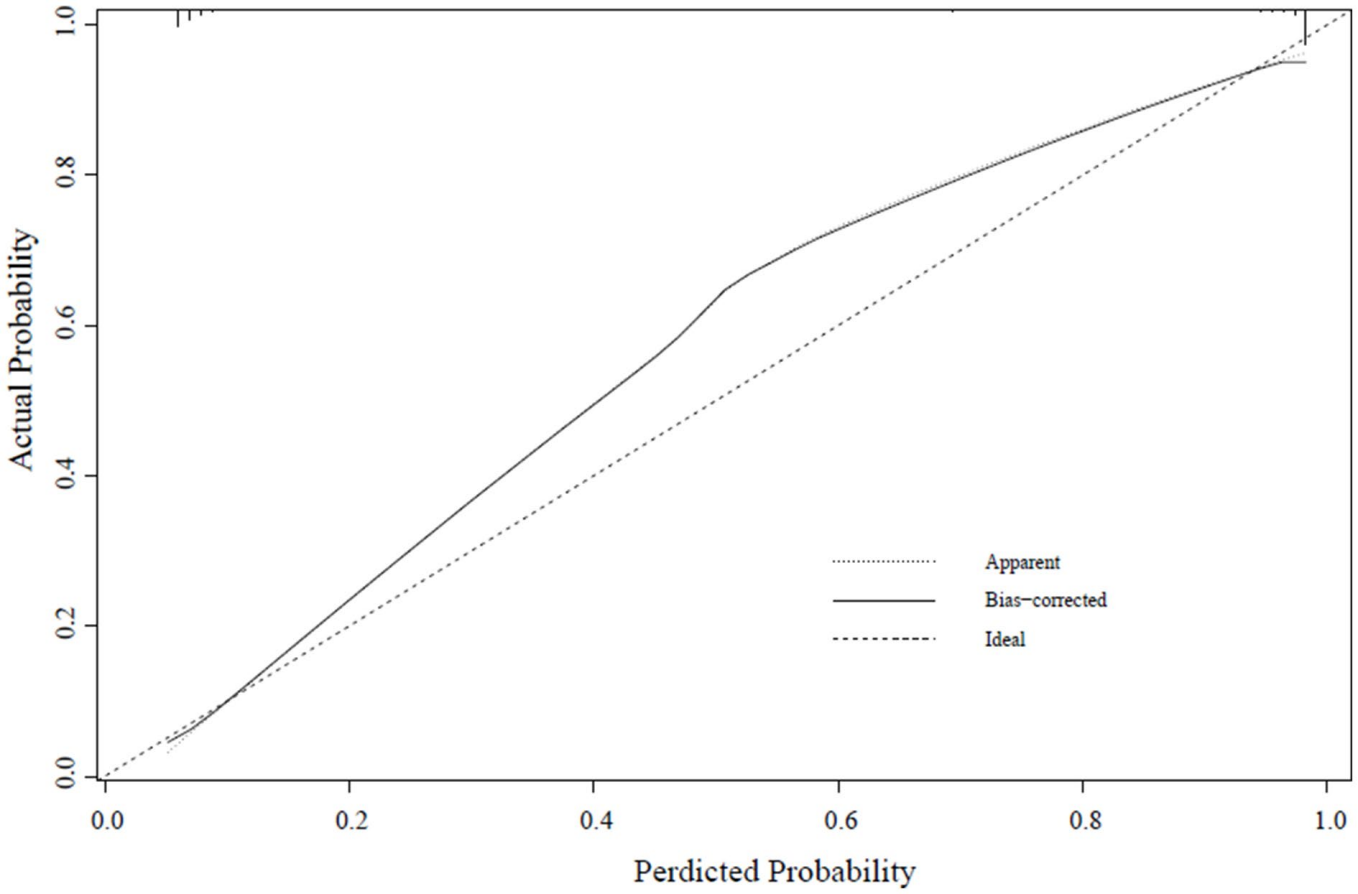
Internal validation set calibration curve.

**Figure 6:**
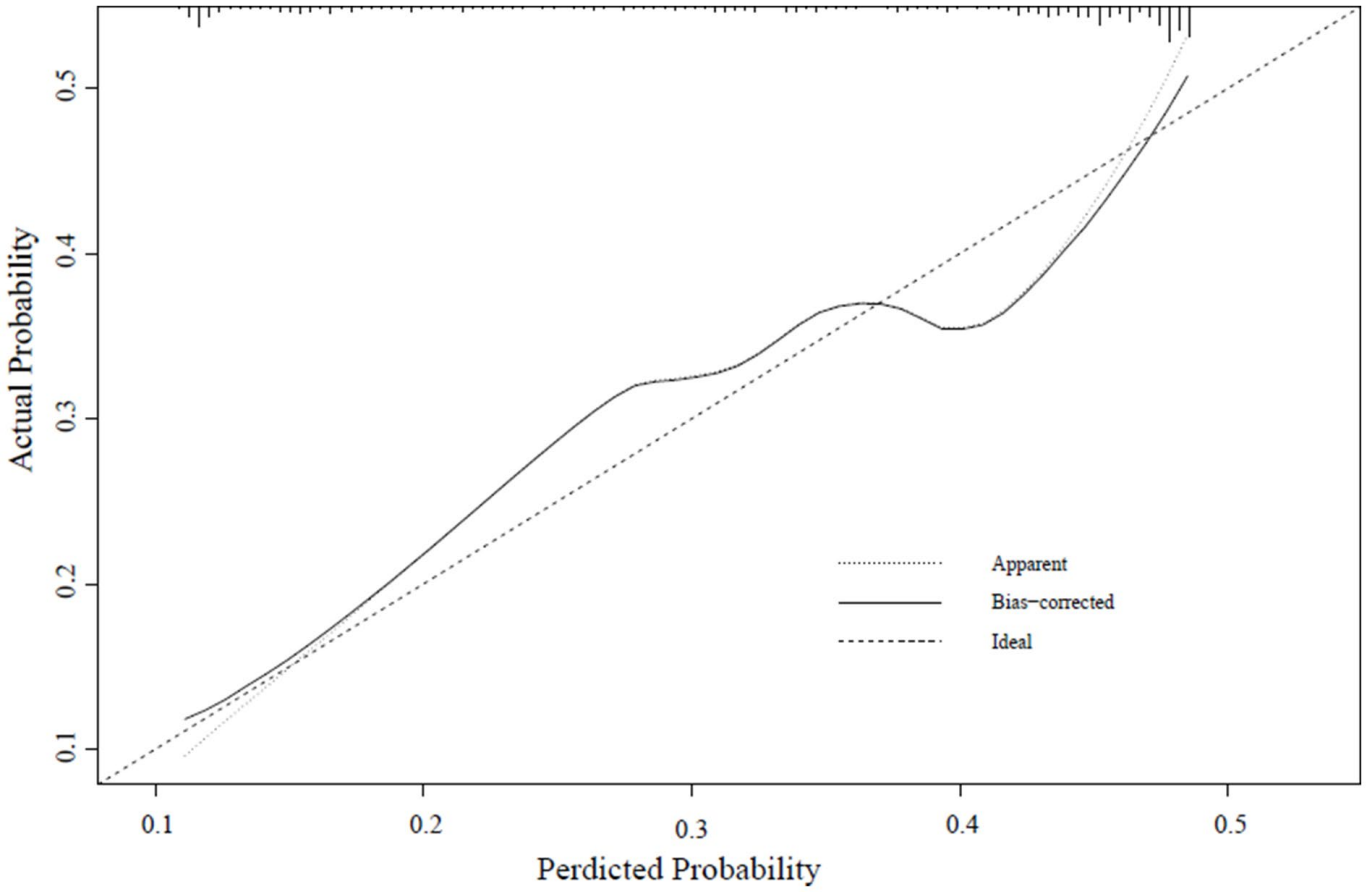
External verification calibration curve.

A calibration curve was constructed to determine the consistency of the logistic regression model (19). The ideal curve aligns closely with the bias-connected curve, indicating excellent calibration of the model (Figures 4–6).

### 3.6 Stratified analyses based on some risk factors

During the modeling process, certain variables had to be excluded due to excessive missing values. However, based on guidelines and numerous previous studies, BMI (20–24), smoking history (3, 25–27), and smoking cessation history (28, 29) may be high-risk factors for COPD development and may play a significant role in the diagnosis and evaluation of COPD. Therefore, in this study, the model was applied across various subgroups of the population. The results indicated that, except for variables with insufficient data for fitting, the model had demonstrated robust predictive capability across populations with or without a history of smoking, different BMI levels, varying smoking cessation histories, ≥40 pack-years of smoking, cessation of smoking at age <65 years, and different percentages of EOS (Table 8).

**Table 8:** Risk stratification of the model.

Subgroup	AUC (95% CI)	Accuracy (95% CI)	Sensitivity (95% CI)	Specificity (95% CI)
Cigarette smoking				
No	0.964 (0.918–1.000)	0.955 (0.889–0.988)	0.974 (0.910–0.997)	0.818 (0.482–0.977)
Yes	0.977 (0.961–0.992)	0.925 (0.883–0.956)	0.952 (0.891–0.984)	0.902 (0.836–0.949)
BMI				
<18.5	1.000 (1.000–1.000)	1.000 (0.891–1.000)	1.000 (0.872–1.000)	1.000 (0.478–1.000)
18.5–23.9	0.978(0.959–0.996)	0.929 (0.877–0.964)	0.955 (0.888–0.987)	0.938 (0.850–0.983)
24–27.9	0.986 (0.968–1.000)	0.938 (0.870–0.977)	0.980 (0.891–0.999)	0.896 (0.773–0.965)
≥28	0.896 (0.764–1.000)	0.800 (0.593–0.932)	0.857 (0.572–0.982)	0.727 (0.390–0.940)
Pack-years of smoking^a^				
<40	–	–	–	–
≥40	0.979 (0.921–1.000)	0.947 (0.740–0.999)	1.000 (0.794–1.000)	0.667 (0.094–0.992)
History of quitting smoking				
Yes	0.975 (0.916–1.000)	0.933 (0.779–0.992)	1.000 (0.872–1.000)	0.333 (0.008–0.906)
No	0.963 (0.903–1.000)	0.949 (0.859–0.989)	0.961 (0.865–0.995)	0.875 (0.473–0.997)
Age of cessation				
<65	0.976 (0.962–0.990)	0.928 (0.892–0.954)	0.959 (0.917–0.983)	0.888 (0.822–0.936)
≥65	–	–	–	–
Eos %				
<2%	0.978 (0.964–0.991)	0.957 (0.914–0.983)	0.893 (0.823–0.942)	0.939 (0.879–0.975)
≥2%	0.953 (0.878–1.000)	1.000 (0.815–1.000)	0.846 (0.546–0.981)	1.000 (0.715–1.000)

- indicates insufficient frequency to fit.

a indicates grouping based on median.

## 4 Discussion

With China’s economic and social development and the increasing aging population, the elderly population is growing rapidly in China. Attention to the health of the elderly has gradually shifted to a greater focus on disease prevention, improving individual function, promoting good health, and prolonging healthy life expectancy. Healthy China 2030 (30) emphasizes that COPD is characterized by high prevalence, disability, mortality, and disease burden.

In this study, we developed a predictive model for COPD using large sample retrospective data, identified four reliable risk factors for COPD, and derived predictive formulas. Following discrimination and calibration tests, the formulas accurately predicted the probability of COPD development within the same sample source while demonstrating average diagnostic effectiveness in external populations.

Age is a significant risk factor for COPD. The higher the age, the greater the prevalence, likely due to age-related decline in lung function and cumulative exposure to environmental pollutants such as tobacco smoke (31). COPD is highly prevalent in individuals aged over 40 years. According to a 2018 study from the Chinese Adult Lung Health Study (3), the prevalence of COPD among individuals aged over 40 years in China was reported to be 13.7%. In our study, the average age of patients with COPD included in the modeling was 70.37 years old. The coefficient of age in the final model was 0.047, indicating a positive correlation between age and COPD risk. This finding reaffirms the demographic distribution characteristics of COPD and underscores the impact of age on its development.

The effects of age on COPD are mainly reflected in the following aspects. First, there is a natural decline in lung function as individuals age. This decline includes reduced respiratory function, decreased alveolar elasticity, thinning of the alveolar wall, and increased airway resistance, leading to the emergence of symptoms such as dyspnea and cough. Second, aging correlates with declining nutritional status (9), impacting food intake and absorption abilities. For patients with COPD, body functions are in a high state of decomposition, leading to increased daily energy expenditure, and a significantly increased risk of malnutrition. Long-term malnutrition leads to muscle atrophy, especially the atrophy of the respiratory muscles, which makes the lungs less compliant and causes a decline in pulmonary ventilation (32). Third, as previously mentioned, aging increases the risk of decreased nutritional status. Without adequate nutrition, the immune system cannot function properly and the risk of lung infection is increased (33). In the elderly, each infection poses a significant threat to lung function, and the resultant damage is difficult to reverse. In patients with COPD, inflammatory irritation of the airways persists, and airways are constantly remodeling (34). Repeated infections exacerbate inflammatory and airway remodeling, further worsening pre-existing airway obstruction.

The primary components of tobacco are tar and nicotine, which cause inflammation, oxidative stress, and apoptosis. Cigarette smoke induces chronic inflammatory responses throughout the body by increasing the levels of inflammatory factors such as IL-1, IL-6, and TNF-*α* (35). The brain is highly sensitive to hypoxia, and cigarette smoke aggravates pulmonary ventilation and hypoxemia (31). This situation further slows cellular metabolism and promotes neuronal apoptosis (36).

COPD is a heterogeneous state of the lungs characterized by persistent airflow obstruction due to airway and/or alveolar abnormalities, often accompanied by chronic cough. Pathological changes in COPD involve the airways, lung parenchyma, and blood vessels. Airway alterations, in particular, play a significant role in causing cough (34), as they sustain persistent inflammation leading to mucus hypersecretion and ciliary dysfunction (37). However, narrowing of the airways makes it difficult to expel sputum in the lungs, which in turn stimulates the airways and causes cough. Many patients with COPD also experience allergic diseases, such as asthma and allergic rhinitis, which heighten airway receptor sensitivity and exacerbate cough due to allergic triggers. Patients with COPD are susceptible to bacterial and viral infections due to decreased immunity, further stimulating the airways to cause coughing.

Wheezing is common in patients with COPD, especially in severe disease or acute exacerbation. This study categorized wheezing into four distinct levels of symptoms to assess its diagnostic utility for mild COPD. The results showed that the different levels of wheezing symptoms were diagnostic factors of COPD, suggesting that the presence of wheezing symptoms holds diagnostic significance for identifying COPD once they manifest.

While several prediction models for COPD have been developed in China, most of them focused on studying risk factors for acute exacerbation and have been conducted within specific medical units or regions. In contrast, the present study is a multi-center clinical study with modeling samples from provincial-level tertiary hospitals in Zhejiang, Jiangxi, and Chengdu. This approach has allowed us to achieve a larger sample size, enhancing the regional representativeness and practical application of our final model. The external validation set utilized data from medical examination centers and health centers affiliated with tertiary hospitals, ensuring sample diversity across a broad spectrum. This approach effectively demonstrates whether our model can be widely applied in clinical settings.

China is a country with a high prevalence of COPD. Although lung function is an important basis for diagnosing COPD, many regions lack the conditions for lung function testing. Therefore, we aim to establish a predictive model that incorporates symptoms and routine biological indicators as much as possible as such a model would have broader application potential. For example, during annual physical examinations, if a doctor assesses that a patient has reached the high-risk threshold predicted by the model, they can refer the patient for pulmonary function testing. Additionally, the model can be used to stratify the risk of COPD among the examined population, thereby better assessing the risk of COPD.

However, the sample for this study is not yet sufficient, especially in terms of external validation specificity. One main limitation is sample selection bias. The retrospective data used to build our model came from three provinces in eastern and southwestern China, but due to the sudden outbreak of COVID-19, we were only able to include external validation data from one province in eastern China, resulting in sample bias, which we deeply regret. Additionally, since we used large-scale retrospective data to build the model, many indicators had to be excluded due to data missingness exceeding 10%, though we still analyzed some indicators we deemed important in risk stratification, which is another contributing factor. This model is suitable for the elderly population, which is one of its limitations. Although the model is biased, it is based on a multicenter design and has undergone rigorous validation, and we believe it still has significant value.

In comparison to a study published in Lancet Respiratory Medicine in 2020 (38), they developed a predictive tool to forecast, at an individual level, the rate and severity of COPD exacerbations, reported on its performance in an independent external cohort, and explained, using case studies, its potential clinical application. In 2022 (39), Thorax published an article using causal machine learning to explore the impact of individualized treatment on COPD exacerbations. These two studies suggest that identifying individual responses to COPD progression, exacerbations, and treatment may be more valuable for clinical diagnosis and management of COPD. This provides significant inspiration for our future COPD research. However, our team has not ceased clinical research on COPD. We continue to enroll COPD patients from different provinces and try to develop a more adaptive predictive model, even a digital diagnostic tool.

## 5 Conclusion

We have developed a predictive model for COPD for clinical use, enabling healthcare professionals, especially those in primary care settings, to quickly and conveniently assess the risk of COPD, thereby promoting timely diagnosis and treatment. However, this model still needs further verification. Until the model is more refined, it is recommended to use it with caution.

## Data Availability

The datasets presented in this article are not readily available because all data are stored in the First Affiliated Hospital of Zhejiang Chinese Medical University, Hangzhou, China. The data used and/or analyzed during the current study can be obtained from corresponding author. However, the data are not publicly available due to privacy or ethical restrictions. Requests to access the datasets should be directed to WZ, wangzhen610@sina.cn.
